# Does a Human-drafted Letter Edited by Chat Generative Pre-Trained Transformer Escape Detection by Artificial Intelligence Detectors?

**DOI:** 10.31662/jmaj.2025-0256

**Published:** 2025-11-14

**Authors:** Shigeki Matsubara

**Affiliations:** 1 Department of Obstetrics and Gynecology, Jichi Medical University, Tochigi, Japan; 2 Department of Obstetrics and Gynecology, Koga Red Cross Hospital, Koga, Ibaraki, Japan; 3 Medical Examination Center, Ibaraki Western Medical Center, Chikusei, Ibaraki, Japan

**Keywords:** artificial intelligence, ChatGPT, detector, edition, letter

## Introduction

Generative artificial intelligence (GenAI, e.g., ChatGPT) use in medical writing attracts attention. For simplicity, I target “letter writing.” When using GenAI, many write their draft first, followed by GenAI editing, adopting a “human-first approach.” Many journals allow GenAI use for “readability refinement” ^(1), (2)^; however, it may change only 1% or even 80% of the initial draft.

The World Association of Medical Editors ^(3)^ states that journals should have artificial intelligence (AI) content detectors to check for excessive GenAI reliance. Then, to what extent does GenAI editing trigger red flags from AI detectors? I do not suggest specific allowable extents; rather, I aim to provide real-world data because knowing the facts is fundamental.

## Methods

### Fundamental study design

**Figure 1.**
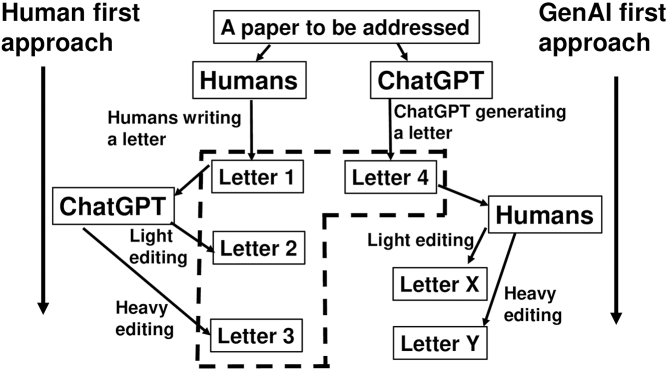
Schematic presentation of the study. The left half illustrates the “human-first” approach, whereas the right half depicts the “GenAI-first” approach. As shown, this study focused on the former approach, with Letter 4 (generated through the GenAI-first approach) used solely as a positive control. The dotted square indicates Letters 1-4, highlighting their relationship to the human-first and GenAI-first approaches.

Figure 1 indicates the schema. I wrote a letter (Letter 1). I tasked ChatGPT-4 with editing “grave mistakes,” resulting in Letter 2. Next, I tasked ChatGPT with “editing the manuscript,” which yielded an extensive edition, making Letter 3. I input the whole paper to be addressed ^(4)^ into ChatGPT and tasked it with “generating a disagreement letter” (Letter 4), serving as a positive control of GenAI generation. In every step, I reset ChatGPT. Five free AI detectors checked AI content probability. Institutional review board approval is not applicable.

### Letter 1: Human-written

The paper described a higher incidence of low birth weight in rural than urban areas ^(4)^, which was attributed not to rural living but to some associated factors. I thought the “intention to live rural” should be considered. I wrote this letter in approximately 60 minutes without performing a linguistic check, leaving room for AI’s edition to create Letters 2 and 3. Letter 1 also serves as a negative control for AI detection.

### Letter 2: ChatGPT’s light edition

I tasked ChatGPT-4 with editing Letter 1, prompting, “Edit Letter 1. Check ‘grave’ errors, while holding my style.” This became Letter 2.

### Letter 3: ChatGPT’s heavy edition

I tasked ChatGPT-4 with editing Letter 1 using only the prompt “Edit Letter 1.” This became Letter 3.

### ChatGPT-generated letter

I inputted into ChatGPT-4 the whole manuscript to be addressed ^(4)^ and tasked it with generating a disagreement letter, also tasking with involving the viewpoint “intention to live where.” This became Letter 4, serving as a positive control. Letters 1-4 are provided in the Supplementary File.

### AI content probability by five AI detectors

I used five free AI content detectors that appeared first in Yahoo search (Table 1). These five detectors calculated AI content probability.

**Table 1.** The AI Content Probabilities of the Four Letters (1-4) and AI Content Detectors Used.

Letter	1	2	3	4
GPTZero	0	0	100	100
Quillbot	0	0	89	60
Isgen	0	0	100	100
Scribbr	0	0	89	60
Surfer	0	0	100	100

Numerals represent the probability (%) of AI generation (see text); 100% indicates that the letter was entirely AI-generated, whereas 0% means it was entirely human-written. GPTzero https://gptzero.me Quillbot https://quillbot.com Isgen https://isgen.ai/ja Scribbr https://www.scribbr.com/ai-detector/ Surfer https://surferseo.com/ai-content-detector/ AI: artificial intelligence.

## Results

Letter 1 may reach a level close to my previously published papers. Letter 2 became refined: I felt I wrote Letter 2 myself. Letter 3 underwent extensive editing not only linguistically but also structurally. I did not feel I wrote it. Letter 4 describes a context similar to Letters 1, 2, and 3; however, I felt Letter 4 was entirely written by somebody else.

Letter 1 (human-written) showed 0% AI content probability, serving as a negative control. Letter 4 (GenAI-generated) showed 60%-100% probability and was fundamentally serves as a positive control. Letter 2, ChatGPT’s minorly edited version, showed 0% probability. Letter 3, extensively edited by ChatGPT, showed 89%-100% probability (Table 1).

## Discussion

The order of “edit” led to a high AI generation probability, up to 100%, whereas the order of “check grave errors while holding my style” did not increase the probability, which remained at 0%. For simplicity, the former and latter are referred to as “heavy” and “light” editing, respectively.

First, a manuscript undergoing ChatGPT’s heavy editing version was judged as highly AI-generated. When authors task GenAI with editing a human-drafted manuscript, the prompt may vary depending on the individual ^(5)^. When I first checked ChatGPT’s editing capacity, I experimentally prompted “edit my manuscript.” I found that this order drastically changed my initial draft, completely erasing my writing style. It did not feel mine. Less-experienced, especially non-native, authors might simply prompt “Edit!,” and ChatGPT will provide a linguistically and structurally complete version. They may be attracted by the beautifully edited paper and may submit it ^(6)^. Even less-experienced authors could easily write a “rough draft” expressing key points and let ChatGPT immediately edit it, generating a well-written letter (e.g., Letter 3). Some cannot resist this temptation: writing only a draft and asking ChatGPT. Setting aside the discussion of whether this procedure is ethically right, we must recognize that it results in a judgment of 100% AI probability.

Second, ChatGPT’s light editing version did not increase AI probability; it remained 0%. I recognized the following prompt fits my purpose: “edit only grave errors, while maintaining my writing style or tone” ^(6), (7)^. This usually generates a linguistically correct manuscript that holds my personal tone. Even when I use ChatGPT, I confine its use to this prompt. Otherwise, although initially starting with this approach, temptation may lead me to ask for more and more editing, losing my tone. Many journals admit ChatGPT use for “readability refinement ^(1), (2)^”. I believe that the use for Letter 2 may fall under this refinement. Letter 3 seems far beyond “refinement.” The point is that editing exists on a spectrum between Letters 2 and 3. Interestingly, AI detectors did not raise a red flag for Letter 2 but did for Letter 3. This does not mean that the demarcation of allowable versus non-allowable ChatGPT use lies between Letters 2 and 3. However, my feelings of allowable versus non-allowable AI use coincided with AI detector not raising versus raising a red flag.

There are some limitations. I drafted Letter 1 myself and did not experiment with letters drafted by others, including native speakers or less-experienced writers, meaning, this study was based on a single author and a single letter. Each condition was tested only once. Examination of variability and reproducibility through repeated experiments has not been made. I did not experiment with manuscript types other than letters. To the best of my knowledge, there is no standard template for tasking ChatGPT with editing, and the present prompt was tentative and may have been too simple. Indeed, as described, a binary distinction between heavy and light editing is difficult. To allow readers to reproduce the experiment, five “free” AI detectors were used; however, it is unclear whether these five were appropriate choices. Indeed, the algorithms of these AI detectors are black boxes, and, thus, confrontation between AI (ChatGPT) and AI (detectors) can occur. Furthermore, only ChatGPT was used, and other large language models were not examined. I also did not experiment with a ChatGPT-first approach: how AI detectors raise red flags to human editing of a ChatGPT-generated manuscript (Figure 1, right row, Letters X and Y) ^(8)^. I also did not analyze which of Letters 1-4 is better. For reference, I included ChatGPT-4’s evaluation (Supplementary File): letters were judged to have similar readability.

Thus, the present humble experiments target limited materials, preventing firm conclusions, which are the study limitations. However, some of these limitations may turn into strengths. I illustrated the most likely scenario: human-first with the prompt “Edit!” Experienced non-native authors were suited to this experiment because one can write a “readable” but “linguistically incomplete” draft. Less-experienced authors or native speakers may struggle to generate such a letter (Letter 1), which is readable but retains “room” for ChatGPT’s light and heavy edits. Regardless, my intention was to provide a platform for discussion on what’s adequate in GenAI use in the human-first approach. I believe I have, at least to some extent, succeeded in this.

This study does not aim to show how to avoid AI detectors or what level of GenAI use is safe. It simply provides real-world data on AI content probabilities after different GenAI edits. How readers use this depends on their judgment.

There are two approaches: human-first or GenAI-first ^(8), (9)^. As described, I recommend the former ^(8)^. In this approach, what I considered fair did not trigger AI detectors, whereas what I felt “too much” did; common sense still holds in the AI era.

To date, there is no worldwide consensus on to what extent large language models should be used in academic writing ^(3)^. Their use may streamline the writing process depending on the situation; there are many positive aspects. However, as far as I am concerned, I believe that ChatGPT use in writing should be minimal ^(10), (11)^. If my belief is right, this study helps us consider what “minimal” means.

## Article Information

### Conflicts of Interest

None

### Author Contributions

Shigeki Matsubara wrote the manuscript.

### Data Availability Statement

All data are described in the text and the supplementary files are freely available.

### Declaration of Generative AI and AI-assisted Technologies in the Writing Process

During the preparation of this work the author used ChatGPT-4 to make ChatGPT-edited (Letters 2 and 3) and -generated manuscript (Letter 4) (Supplementary File). The author also used ChatGPT-4 to evaluate Letters 1-4 (Supplementary File). I wrote this in the text and the author takes full responsibility for this. The author did not edit ChatGPT-generated parts because this study compares human-written versus ChatGPT-generated manuscripts.

## Supplement

Supplementary Material Letters 1-4 and ChatGPT’s evaluation of these letters.
