# Supplementary material for: Does a Human-drafted Letter Edited by Chat Generative Pre-Trained Transformer Escape Detection by Artificial Intelligence Detectors?
Source: JMA J. 2025 Nov 14;9(1):389–92. doi: 10.31662/jmaj.2025-0256 (PMC12889295; doi:10.31662/jmaj.2025-0256)
Supplement: Supplementary Material — Letters 1-4 and ChatGPT’s evaluation of these letters. [file 2433-3298-9-1-0389-s001.pdf]

Supplementary File: Letters 1-4 and ChatGPT's evaluation of these four letters

1) Letters 1-4

Letter 1: Low birth weight: due to living rural or intention to live rural?

Britten et al. demonstrated that the incidence of low birth weight (LBW) was higher in rural than in urban areas; however, after adjusting for confounders, this tendency disappeared. Not rural residency itself but associated factors (e.g., smoking habits) caused this higher incidence. They concluded that resources should be devoted to addressing such risk factors: for example, "stop smoking" campaign may be effective. Many studies comparing rural versus urban so far employed this study design: extracting risk factors and proposed targeted policies for such risks.

I wish to propose a new concept. The factor of "intention/choice to live in a rural area" should be incorporated into the study, which may strongly affect the actual living area.

In some people, some factors (typically their jobs) determine the living area, depriving them of the freedom of living area. However, theoretically, many can "choose" where to live. Tokyo residents may enjoy museums and abandon the quiet surf sound. Islander residents may feel the opposite. Where to live depends on one's philosophy regarding what one prioritizes. Thus, the "wish to live in a rural or urban area" determines, at least to some extent, the actual place of living. Some stop smoking. Similarly, some may stop living in rural and move to urban to seek better medical service. "Did you choose to live in a rural area, and do you like it?" This is only an example, but such questionnaires may provide some important information.

Britten's study adhered to the standard clinical study design and was well-structured. If one may design a study incorporating "intention," should it be considered as a confounding factor similar to smoking habits? Intention is difficult to measure. Thus, in-depth knowledge of statistics and study design is necessary. Putting this technical issue aside, I believe incorporating the personal "intention" into analysis may provide some new insight into studies comparing rural versus urban. This holds true beyond obstetrics-gynecology study, let alone an LBW study.

More generally, Britten et al.'s study let me contemplate how personal "intention" affects human health and how we incorporate it to clinical studies. The answer is not easy, but is worth pursuing.

Letter 2: Low birth weight: due to living rural or intention to live rural?

Britten et al. demonstrated that the incidence of low birth weight (LBW) was higher in rural than in urban areas; however, after adjusting for confounders, this tendency disappeared. Not rural residency itself but associated factors (e.g., smoking habits) caused this higher incidence. They concluded that resources should be devoted to addressing such risk factors: for example, a "stop smoking" campaign may be effective. Many studies comparing rural versus urban so far employed this study design: extracting risk factors and proposing targeted policies for such risks.

I wish to propose a new concept. The factor of "intention/choice to live in a rural area" should be incorporated into the study, which may strongly affect the actual living area.

In some people, some factors (typically their jobs) determine the living area, depriving them of the freedom to choose their location. However, theoretically, many can "choose" where to live. Tokyo residents may enjoy museums and abandon the quiet surf sound. Islander residents may feel the opposite. Where to live depends on one's philosophy regarding what one prioritizes. Thus, the "wish to live in a rural or urban area" determines, at least to some extent, the actual place of living. Some stop smoking. Similarly, some may stop living in rural areas and move to urban areas to seek better medical services. "Did you choose to live in a rural area, and do you like it?" This is only an example, but such questionnaires may provide some important information.

Britten's study adhered to the standard clinical study design and was well-structured. If one were to design a study incorporating "intention," should it be considered a confounding factor similar to smoking habits? Intention is difficult to measure. Thus, in-depth knowledge of statistics and study design is necessary. Putting this technical issue aside, I believe incorporating the personal "intention" into the analysis may provide new insights into studies comparing rural versus urban. This holds true beyond obstetrics-gynecology studies, let alone an LBW study.

More generally, Britten et al.'s study led me to contemplate how personal "intention" affects human health and how we incorporate it into clinical studies. The answer is not easy, but it is worth pursuing.

Letter 3: Reevaluating the Link Between Low Birth Weight and Rural Residency: The Role of Personal Choice

Britten et al.'s study underscores an important observation: the incidence of low birth weight (LBW) is higher in rural areas compared to urban centers. However, after adjusting for confounders, this association was no longer statistically significant. This suggests that the higher rates of LBW in rural areas are not inherently due to geographical location, but rather to associated factors such as smoking habits. The authors rightly advocate for targeted interventions, such as smoking cessation programs, to address these risk factors.

While this approach is valid, I would like to offer an alternative perspective—one that could deepen our understanding of the rural-urban divide in health outcomes. Specifically, I believe the concept of intention or choice to live in a rural area merits inclusion in studies of this nature. It is not only the actual place of residence that matters but also the personal decision-making process that leads individuals to settle in rural or urban environments.

For some people, the decision about where to live is driven by external factors, such as employment, which limits their freedom of choice. However, for many others, the choice of residence reflects personal preferences, values, and lifestyle priorities. Just as some individuals might choose to quit smoking to improve their health, others may choose to move from rural areas to urban centers in search of better healthcare and resources. Therefore, the intention to live in a rural area could be a key factor influencing health outcomes, including LBW rates.

To explore this, a study could ask a seemingly simple question: *"Did you choose to live in a rural area, and do you enjoy living there?"* While this may seem a subtle distinction, it could reveal important insights into the relationship between residential location and health outcomes.

Britten et al.'s study adhered to a well-established clinical research design, which is commendable. However, I propose that future studies consider "intention" as an additional variable, akin to smoking habits, that could act as a confounder. Intention is inherently difficult to quantify, but with advanced statistical techniques and careful study design, this variable could yield valuable insights into the rural-urban health divide.

Letter 4: Living Rural, Intending Rural: Unpacking an Overlooked Variable in Maternal Outcomes

I was intrigued by the article titled *"Does Maternal Location of Residence Affect Low Birth Weight Outcomes in Hunter New England Local Health District?"* by Britten et al. The study highlights important insights into the connection between maternal geographical location and low birth weight (LBW), particularly the higher rates in rural maternal residents. The authors make a compelling case for the need for targeted healthcare interventions to address these disparities.

However, one aspect that may further enrich the discussion is the *intention* to live in rural areas, which could have significant implications for understanding LBW outcomes. The choice or intention to live in rural areas, rather than simply the actual residency, may influence a range of health behaviors and access to care. Women who *intend* to live in rural areas might have different motivations, socio-economic backgrounds, or healthcare needs, which could indirectly affect LBW outcomes. These intentions could also shape their healthcare utilization patterns and overall pregnancy care.

While the current study focuses on actual maternal residence, considering a woman's intention to live in a rural location could add another layer of complexity. This distinction, while difficult to measure, could provide valuable insights into the factors influencing LBW beyond just the geographical location. Future research could explore this concept, offering a deeper understanding of how

the *intended* location of residence might relate to maternal and neonatal outcomes, especially in the context of rural health disparities.

I commend the authors for their thoughtful work and look forward to further investigations that incorporate these considerations, which could lead to more targeted and effective health interventions for rural populations.

2) ChatGPT's evaluation of the four letters

Input: The whole manuscript to be addressed [4] and Letters 1-4 (above).
Prompt: "Please give me your opinion on which Letter to the Editor is most likely to be accepted by the journal. Rank them in order (e.g., 1 > 2 > 3 > 4). Also, tell me whether the differences between them are large or slight."

Output (ChatGPT-4's evaluation)

My ranking: 1 > 3 > 2 > 4

Rationale:

- **Letter 1** is most likely to be accepted. It balances originality, clarity, and your signature writing style (thoughtful and slightly philosophical). It's direct yet personal, introduces the new concept well and ends strongly.
- **Letter 3** is more polished in English and more academic in tone but slightly loses your unique voice. It reads like a professional Western-trained letter, which may appeal broadly but lacks the subtle charm of 1.
- **Letter 2** is close to 1 in content but slightly less crisp in phrasing and flow. It reads more neutral and explanatory and less impactful.
- **Letter 4** is the most generic and polite but weak in originality and voice. It sounds more like a reader's compliment than a letter adding new insight.

All the letters are well written, and the differences are minor. Which one will be accepted depends on the editors' preference.
